# Applications of Artificial Intelligence for Static Poisson's Ratio Prediction While Drilling

**DOI:** 10.1155/2021/9956128

**Published:** 2021-05-04

**Authors:** Ashraf Ahmed, Salaheldin Elkatatny, Ahmed Alsaihati

**Affiliations:** College of Petroleum Engineering and Geosciences, King Fahd University of Petroleum and Minerals, Dhahran 31261, Saudi Arabia

## Abstract

The prediction of continued profile for static Poisson's ratio is quite expensive and requires huge experimental works, and the discontinuity in the measurement and the limited applicability and accuracy of the present empirical correlations necessitated the utilization of artificial intelligence with its prosperous application in oil and gas industry. This work aims to construct different artificial intelligence models for predicting static Poisson's ratio of complex lithology at real time during drilling. The functional networks (FN) and random forest (RF) approaches were utilized using the mechanical drilling parameters as inputs. This study uses a vertical well with 1775 records from complex lithology containing shale, sand, and carbonate for model building. Besides, a different dataset from another well was used to check the models' validity. The results demonstrated that both FN- and RF-based models predicted static Poisson's ratio with significant matching accuracy. The FN technique results' correlation coefficient (*R*) value of 0.89 and average absolute percentage error (AAPE) values of 10.23% and 10.28% in training and testing processes. While the RF technique is outperformed, as illustrated by the highest *R* values of 0.99 and 0.94 and the lowest AAPE values of 1.89% and 5.19% for training and testing processes, the robustness and reliability of the developed models were confirmed in the validation process with *R* values of 0.94 and 0.86 and AAPE values of 11.23% and 5.12% for FN- and RF-based models, respectively. The constructed models developed a basis for inexpensive static Poisson's ratio prediction in real time with significant accuracy.

## 1. Introduction

Poisson's ratio (*ν*) is an elastic geomechanical characteristic defined as the ratio between transversal and axial strains. It measures the rock's ability to recuperate from a deformation resulted by external strains and defines the relationship between these strains and the caused deformation [1]. Knowing Poisson's ratio with the other rock elastic properties helps design hydraulic fracturing and interpret the in situ stresses, drilling performance, and wellbore stability [2–5], which help in optimizing the drilling operations, well planning, and development of completion and production strategies.

In practical, Poisson's ratio varies from 0.0 to 0.5 and has two types: static (*ν*_*st*_) and dynamic (*ν*_*dyn*_). The static is determined experimentally from compressional tests, while the dynamic derived from the shear and compressional wave velocities from well logs. The high expenses and discontinuity in the *ν*_*st*_ measurement necessitated its prediction using empirical relationships. Different correlations were developed to predict the *ν*_*st*_ using *ν*_*dyn*_ or compressional and/or shear wave velocities (*V*_*p*_ and *V*_*s*_) with different empirical coefficients, as summarized in Table 1.

The abovementioned empirical correlations have reliability limitations and depend on the availability of *V*_*P*_ and *V*_*S*_. Since the artificial intelligence (AI) can be applied for inexpensively analysing and interpreting tremendous data and trends to obtain outputs in least time [9], it can be utilized to predict a continued profile of Poisson's ratio.

The AI techniques became a hotspot in petroleum industry since the early nineties and applied in different aspects [10, 11]. The AI was applied in exploration to predict the feature recognition [12], travel time calculation [13, 14], and clarity and resolution of seismic data enhancement [15]. Several AI models were applied in drilling aspects such as operations monitoring, data correction, and prediction of drilling parameters and mud properties [16–25]. Also, in reservoir and production engineering, the AI was applied in prediction of reservoir characteristics, inflow performance, production rate, and others [26–38].

Different AI models were developed to estimate the *ν*_*st*_, using the artificial neural network (ANN), support vector machine (SVM), alternating conditional expectation (ACE), fuzzy logic (FL), functional networks (FN), and adaptive neuro-fuzzy inference system (ANFIS), as listed in Table 2.

From a practical point of view, the drilling parameters, such as weight on bit (WOB), standpipe pressure (SPP), torque (*T*), rotation speed (RPM), pumping rate (*Q*), and rate of penetration (ROP) are linked to the formation features and measured at real time [48–50], Consequently, these mechanical parameters can be correlated to *ν*_*st*_ as a geomechanical rock property.

This work aims to construct different models to predict real-time static Poisson's ratio (*ν*_*st*_) of complex lithology during drilling. The functional networks (FN) and random forest (RF) approaches were applied, and the drilling parameters including WOB, *T*, SPP, RPM, ROP, and *Q* were used as inputs. In the following sections, the datasets are described, analysed, and preprocessed. Then, the outputs of the developed models are presented and validated using different datasets.

## 2. Methodology

### 2.1. Data Description

Different datasets were obtained from an intermediate section with a 12.25” hole diameter of two vertical wells having penetrating formations with complex lithology of sand, shale, and carbonate. These datasets contained the real-time drilling parameters, including the WOB, *T*, SPP, RPM, ROP, and *Q*, which were measured at the surface while drilling and the corresponding *ν*_*st*_ from core experiments and conventional logs. The drilling parameters were used as inputs in the models, and the output is *ν*_*st*_. The dataset from the first well containing more than 1775 records is used to construct the models, while the other well's dataset with 762 records is only used to validate the developed models.

### 2.2. Data Analysis and Processing

The obtained data was studied and processed before using the AI approaches to examine the data quality, reliability, and representation. Statistics of the drilling parameters and *ν*_*st*_ are outlined in Table 3. An extensive range of mechanical drilling parameters and *ν*_*st*_ with good data distribution coverage were indicated from the statistics.

**Figure 1:**
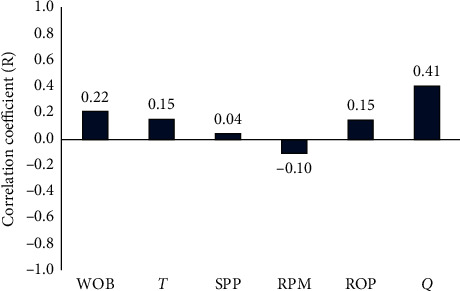
The correlation coefficient between static Poisson's ratio and drilling parameters.

Moreover, the linear relationship strength between the drilling parameters and *ν*_*st*_ was addressed by defining the correlation coefficient (*R*) to measure the relative significance of the drilling parameters for *ν*_*st*_, as illustrated in Figure 1. Although the correlation coefficient between *ν*_*st*_ and SPP indicated almost no linear relation, the SPP varied significantly with changes of *ν*_*st*_. The same behaviour depicted the other parameters; nonlinear relation may exist. Accordingly, all drilling parameters will be considered as inputs.

Data filtration and cleaning were conducted to remove impractical values, noises, and outliers, which improve the data quality and confidence. The tools' limitations, feasible ranges of drilling parameters, and statistical analyses were accounted in these preprocesses.

### 2.3. Models' Development

The cleaned data was divided into 70/30% (1243/532 datapoints) to train/test the models on a random basis. The FN- and RF-supervised learning AI techniques were utilized using Python software with the Scikit-Learn® framework. Sensitivity analyses and tuning process were performed to determine the optimum parameters for each model. Then, the constructed models were evaluated using the data records from the second well.

The FN and RF techniques were nominated for using since they were recently applied in oil and gas industry [34, 44, 51] with encouraging outputs and simplicity.

Multiargument functional models are used in the FN approach to learn and process from the data. This technique relies on both data knowledge and domain. The model's accuracy depends on selecting the best method and type in the FN algorithm. It included five methods (i.e., forward selection (FS), exhaustive search (ES), backward-forward (BF), forward-backward (FB), and backward elimination (BE)) that were tested with the two types of linear and nonlinear to build the optimal model.

On the contrary, the RF technique depends on bagging and bootstrapping procedures. Numerous decision trees are combined and decorrelated, and these processes are performed by randomly choosing parameters at each candidate split. The optimal model parameters of decision trees' number (n_estimators), depth of each tree (max_depth), minimum number of samples desired to divide an internal node (min_samples_split), minimum sample leaf size required to be at the end node (min_sample_leaf), and maximum number of features (max_features) were defined using the tuning process.

The optimality of each model was examined for the highest *R* and lowest AAPE values. The equations for *R* and AAPE calculations are presented in Appendix A.

**Figure 2:**
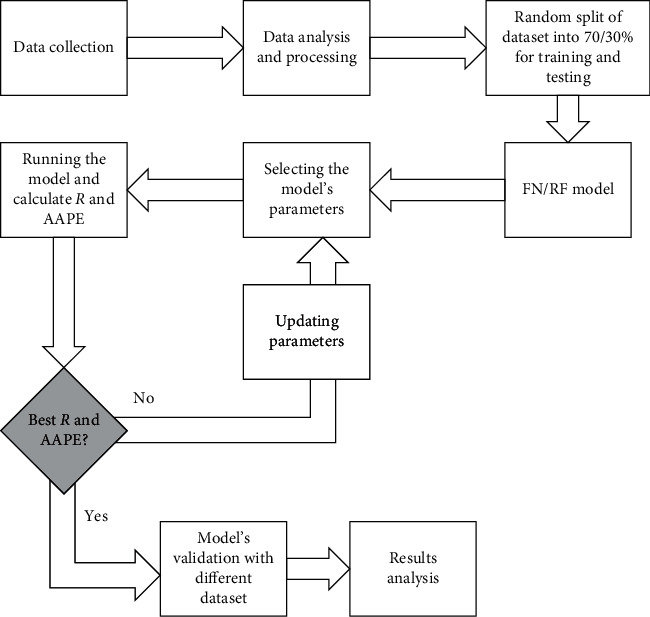
Flowchart of the FN/RF model development.

The flowchart for the methodology of developing the FN/RF is shown in Figure 2.

## 3. Results and Discussion

### 3.1. FN Model

**Figure 3:**
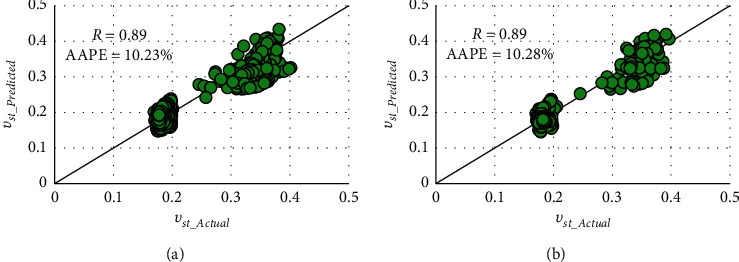
Cross plots of actual and FN-based predicted static Poisson's ratio in (a) training and (b) testing.

**Figure 4:**
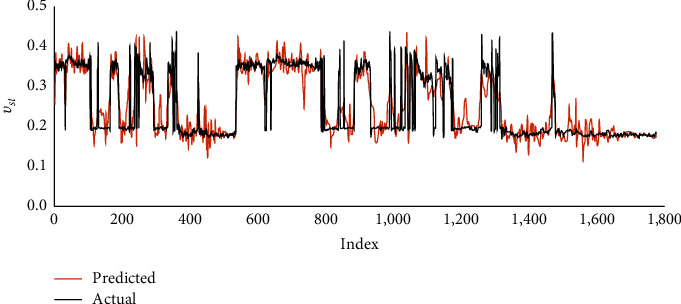
Graphical representations of actual and FN-based predicted static Poisson's ratio.

The backward-forward (BF) method with the nonlinear type resulted the highest matching accuracy specified by the *R* value of 0.89 and AAPE values of 10.23% and 10.28% in training and testing processes. Figures 3 and 4 describe the matching in the cross plots and graphical representations of predicted and actual *ν*_*st*_, respectively.

### 3.2. RF Model

The parameters' tuning process indicated that the superior RF model was acquired using the optimized parameters mentioned in Table 4.

**Figure 5:**
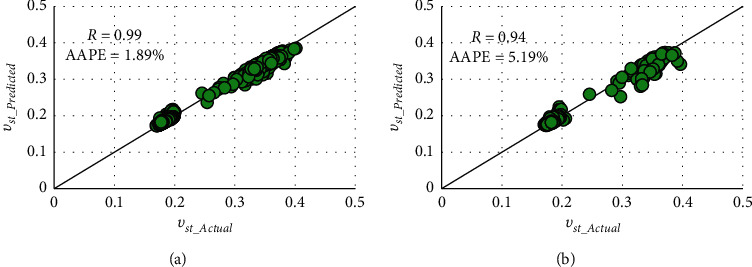
Cross plots of actual and RF-predicted static Poisson's ratio in (a) training and (b) testing.

**Figure 6:**
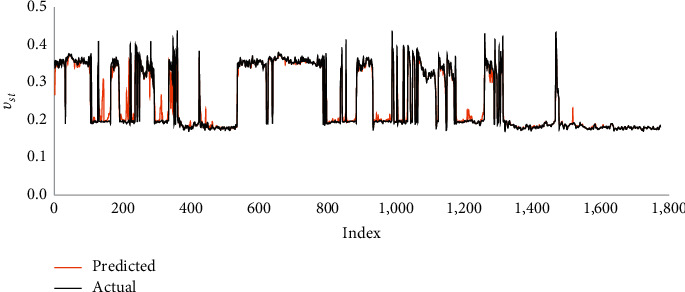
Graphical representations of actual and RF-based predicted static Poisson's ratio.

The developed RF model presented high matching accuracy with *R* values of 0.99 and 0.94 and AAPE values of 1.89% and 5.19% for processes of training and testing. The significant fitting was indicated by the cross plots and graphical representations of predicted and actual *ν*_*st*_, as presented in Figures 5 and 6.

### 3.3. Models' Validation

The reliability of the built models was evaluated using the second well's dataset. This set included 762 records with drilling parameters and corresponding *ν*_*st*_.

**Figure 7:**
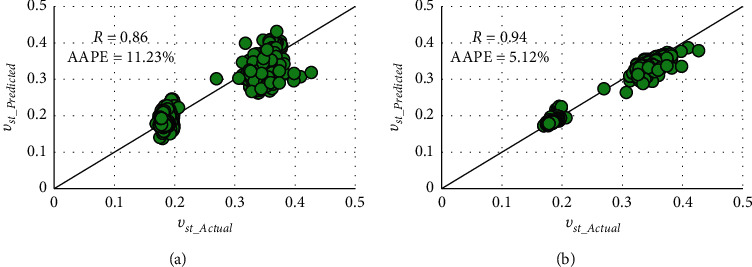
Cross plots of actual and predicted static Poisson's ratio of the validation dataset in (a) FN and (b) RF.

**Figure 8:**
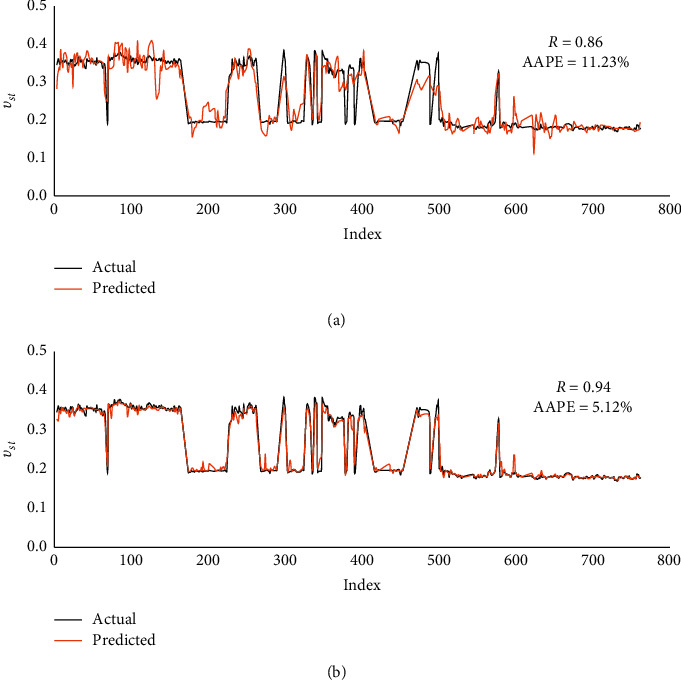
Graphical representations of actual and predicted static Poisson's ratio of the validation dataset in (a) FN and (b) RF.

The obtained results with the validation dataset indicated good matching accuracy with *R* values of 0.86 and 0.94 and AAPE values of 11.23% and 5.12% for FN- and RF-based models, respectively. These results assured the robustness of the developed models and the superiority of the RF model. The cross plots and graphical representations of the predicted and actual *ν*_*st*_ are depicted in Figures 7 and 8.

## 4. Discussion

This paper treated the interest of using the AI approaches for *ν*_*st*_ prediction, to reduce the costs of experiments and overcome the missed data issue for providing a continued profile of *ν*_*st*_. A real field data was used for this scientific and professional research.

An iterative approach was applied to find the optimal parameters of each model. From that, the highest fitting in the RF model was acquired with 100 decision trees, depth of each tree as 15, square root option for maximum number of features, min_samples_split as 2, and one minimum sample leaf size, while the backward-forward method with the nonlinear type was the optimum parameters for the FN approach.

The optimality of the two models was evaluated according to the *R*, AAPE, cross plots, and graphical representations. As a result, both models predicted the real-time *ν*_*st*_ with outperformance in the RF-based model.

As it is impractical to predict a continued profile of *ν*_*st*_ from experiments because of the high expenses, the constructed models are able to estimate *ν*_*st*_ in real time with the least errors. However, the shortness of the developed models was represented by the inputs and outputs' range, as only the intermediate hole section was investigated in these models, so wider lithologies should be examined. Also, the application of the hybrid model to improve the selection of models' parameters instead of iterative methods is recommended for further works as well as the use of other AI techniques.

**Figure 9:**
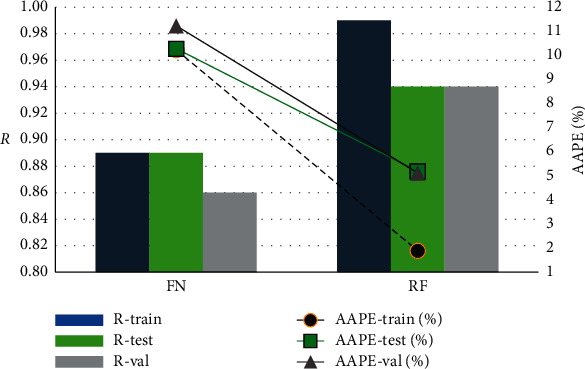
Comparison between FN- and RF-based models.

Comparing the two models indicated that the RF approach had an outperformance in predicting the real-time *ν*_*st*_ with better *R* and AAPE values, as presented in Figure 9. The fitting indices for the two models were outlined in Table 5.

## 5. Conclusions

Two AI models using FN and RF techniques were provided for real-time prediction of static Poisson's ratio from the drilling parameters (WOB, RPM, SPP, *T*, ROP, and Q). A vertical well containing complex lithology with carbonate, shale, and sand was used to build the models utilizing the real field measurements. The main deliverables of this work are summarized as follows:

- The FN model resulted acceptable fitting with the *R* value of 0.89 and AAPE values of 10.23% and 10.28% in training and testing processes
- The RF-based model outperformed in both training and testing processes with *R* values of 0.99 and 0.94 and AAPE values of 1.89% and 5.19%
- The models were validated with a different dataset from another well confirming the models' applicability as it resulted in good prediction fitting with *R* values of 0.86 and 0.94 and AAPE values of 11.23% and 5.12% for FN and RF models, respectively
- Since the experimental measurements of static Poisson's ratio is highly expensive and not always available, the constructed models developed a basis for predicting a continuous profile at real time in inexpensive methods with the least errors

## Figures and Tables

**Table 1:** Empirical correlations for static Poisson's ratio prediction.

Authors	Equations	Remarks
Christaras et al. [6]	*ν*_*st*_=0.71 *ν*_*dy* *n*_+0.063	A correlation between *ν*_*st*_ and *ν*_*dyn*_ was proposed using 8 samples from several rock kinds (i.e., gypsum, phonolite, basalts, granite, limestone, and andesite) The correlation coefficient (*R*) of this model in the specified rock types is 0.737
Feng et al. [7]	*ν*_*st*_=*a* *ν*_*dy* *n*_ − *b*	The same approach was followed to obtain a linear correlation between *ν*_*st*_ and *ν*_*dyn*_ using 18 samples from sandstone and siltstone rocks The empirical coefficients vary with the porosities The coefficient of determination (*R*^2^) is 0.92 and 0.7 for modelling and testing samples, respectively
Wang et al. [8]	*ν*_*st*_=*a* *V*_*p*_+*bν*_*st*_=*c* *V*_*s*_+*d*	*V*_*P*_ and *V*_*S*_ were used to develop two correlations for *ν*_*st*_ prediction at different rocks The empirical parameters change with different rock types The *R* values range from 0.467–0.834 and 0.668–0.914 for the two models, respectively

**Table 2:** The developed AI models for static Poisson's ratio prediction.

Authors	Inputs	AI techniques	No. of datapoints	Remarks
Abdulraheem et al. [39]	Travel time and bulk density	ANN, FL, and FN	77	*R* = 0.10–0.91
Al-anazi et al. [40]	Bulk density, depth, pore pressure, overburden stresses, minimum horizontal stresses, porosity, and compressional and shear travel times	ACE	602	*R* = 0.997
Tariq et al. [41]	*V*_*p*_ and *V*_*s*_	ANN	550	Carbonate formations *R* = 0.985
Elkatatny et al. [42]	Bulk density and compressional and shear times	ANN	610	Carbonate formations *R* = 0.985
Elkatatny [43]	Sonic travel times and bulk density	ANN, ANFIS, and SVM	610	Carbonate formations *R* = 0.933–0.985
Tariq et al. [44]	Bulk density, gamma ray, porosity, and *V*_*p*_ and *V*_*s*_	FN	580	Carbonate formations *R* = 0.985
Abdulraheem [45]	*V*_*p*_ and *V*_*s*_	ANN and FL	75	Carbonate formations AAPE^*∗*^ = 5.16–8.20%
Gowida et al. [46]	Bulk density and sonic log	ANN coupled with DE^*∗∗*^	692	Sandstone *R* = 0.964
Ahmed et al. [47]	Drilling parameters	ANN, ANFIS, and SVM	1775	*R* = 0.90–0.96

^*∗*^AAPE = average absolute percentage error. ^*∗∗*^DE = differential evolution algorithm.

**Table 3:** Statistics of the obtained dataset.

Parameter	WOB (klb_m_)	T (klb_f_.ft)	SPP (psi)	RPM	ROP (ft/hr)	Q (gal/min)	*ν*_*st*_
Minimum	1.54	4.55	2140.20	77.94	27.41	697.31	0.17
Maximum	25.48	10.68	3075.56	162.49	119.57	854.01	0.43
Mean	15.39	7.83	2634.51	138.62	76.39	803.07	0.25
Mode	1.54	4.55	2140.20	77.94	27.41	697.31	0.17
Median	16.27	8.25	2685.02	139.15	79.90	809.77	0.20
Standard deviation	6.44	1.65	205.33	11.08	19.15	48.38	0.08
Skewness	-0.37	-0.37	-0.78	-1.98	-0.55	-1.19	0.50
Kurtosis	2.15	1.91	2.53	10.81	2.41	3.17	1.39

**Table 4:** Optimum set of parameters for the RF model.

Parameter	Value
n_estimators	100
max_depth	17
max_features	Sqrt
min_samples_split	2
min_samples_leaf	1

**Table 5:** The fitting indices for the constructed models.

	*R*			AAPE, %		
	Training	Testing	Validation	Training	Testing	Validation
FN	0.89	0.89	0.86	10.23	10.28	11.23
RF	0.99	0.94	0.94	1.89	5.19	5.12

## Data Availability

All data used to support the findings of the study are available within the article and can be obtained from the corresponding author upon request.

## Appendix

The *R* and AAPE values used to evaluate the optimality of each model are calculated using the following equations:

(A.1) $$\begin{array}{c} R=\frac{\left[N\sum \left(\upsilon_{\text{st\_Actual}}\times \rho_{\text{b\_Predicted}}\right)\right]-\left[\sum \upsilon_{\text{st\_Actual}}\times \sum \upsilon_{\text{st\_Predicted}}\right]}{\sqrt{\left[N\sum {\left(\upsilon_{\text{st\_Actual}}\right)}^{2}-{\left(\sum \upsilon_{\text{st\_Actual}}\right)}^{2}\right]\left[N\sum {\left(\upsilon_{\text{st\_Predicted}}\right)}^{2}-{\left(\sum \upsilon_{\text{st\_Predicted}}\right)}^{2}\right]}}, \end{array}$$

(A.2) $$\begin{array}{c} \text{AAPE}=\frac{\sum^{}\left|\upsilon_{st\_Actual}-\upsilon_{st\_Pre\;di\;cte\;d}/\upsilon_{st\_Actual}\right|\times 100}{N}, \end{array}$$

where *ν*_st_Actual_ and *ν*_st _Predicted_ are the measured and the model-based predicted static Poisson's ratio, respectively and *N* represents the number of datapoints.

## Abbreviations

AAPE: Average absolute percentage error
ACE: Alternating conditional expectation
AI: Artificial intelligence
ANFIS: Adaptive neuro-fuzzy inference system
ANN: Artificial neural network
BE: Backward elimination
BF: Backward-forward
DE: Differential evolution algorithm
ES: Exhaustive search
FB: Forward backward
FL: Fuzzy logic
FN: Functional networks
FS: Forward selection
max_depth: Depth of decision tree
max_features: Maximum number of features
min_sample_leaf: Minimum sample leaf size required to be at the end node
min_samples_split: Minimum number of samples required to split an internal node
n_estimators: Number of decision trees
ROP: Rate of penetration, ft/hr
RPM: Rotation speed, rotation per minute
SPP: Standpipe pressure, psi
SVM: Support vector machines
T: Torque, klb_f_.ft
WOB: Weight on bit, klb_m_

## Symbols

*a, b, c,* and *d*: Different empirical constants
*N*: Number of datapoints
*Q*: Pumping rate, gal/minute
*R*: Correlation coefficient
*V*_*P*_: Compressional-wave velocity, km/s
*V*_*S*_: Shear-wave velocity, km/s
*V*_*Sh*_: Shale volume factor
*ν*_*dyn*_: Dynamic Poisson's ratio
*ν*_*st*_: Static Poisson's ratio.
